# Looking back on a decade of barcoding crustaceans

**DOI:** 10.3897/zookeys.539.6530

**Published:** 2015-11-23

**Authors:** Michael J. Raupach, Adriana E. Radulovici

**Affiliations:** 1 Molecular Taxonomy of Marine Organisms, German Centre of Marine Biodiversity Research (DZMB), Senckenberg am Meer, Südstrand 44, 26382 Wilhelmshaven, Germany; 2 Biodiversity Institute of Ontario (BIO), University of Guelph, 50 Stone Road E, Guelph (ON) N1G 2W1, Ontario, Canada

**Keywords:** Barcode of Life Data Systems, Crustacea, cytochrome *c* oxidase subunit I, DNA barcoding, mitochondrial DNA, specimen identification

## Abstract

Species identification represents a pivotal component for large-scale biodiversity studies and conservation planning but represents a challenge for many taxa when using morphological traits only. Consequently, alternative identification methods based on molecular markers have been proposed. In this context, DNA barcoding has become a popular and accepted method for the identification of unknown animals across all life stages by comparison to a reference library. In this review we examine the progress of barcoding studies for the Crustacea using the Web of Science data base from 2003 to 2014. All references were classified in terms of taxonomy covered, subject area (identification/library, genetic variability, species descriptions, phylogenetics, methods, pseudogenes/numts), habitat, geographical area, authors, journals, citations, and the use of the Barcode of Life Data Systems (BOLD). Our analysis revealed a total number of 164 barcoding studies for crustaceans with a preference for malacostracan crustaceans, in particular Decapoda, and for building reference libraries in order to identify organisms. So far, BOLD did not establish itself as a popular informatics platform among carcinologists although it offers many advantages for standardized data storage, analyses and publication.

## Introduction

The accurate diagnosis of species represents a pivotal component for many topics, including large-scale biodiversity studies and conservation planning. Traditionally, species are identified using morphological characters. This approach requires a certain level of training in observing morphology and it usually leads to a narrow specialization in identifying organisms belonging to a restricted group of taxa (e.g. a carcinologist will likely have difficulties in identifying polychaetes and the other way around). Therefore, a routine and correct morphological identification of many taxa can be challenging, time-consuming and typically requires highly trained specialists. This is especially true for larval stages, juveniles and females which are often not included in species descriptions, resulting in a quite difficult task of assigning correct species names to specimens. In many cases morphological variability and phenotypic plasticity may also complicate a correct species determination. Furthermore, we observe a decline of taxonomists that are able to identify and characterize species of many taxa (e.g. de Carvalho et al. 2007).

As consequence of the rise of molecular biology in the last decades, the application of DNA sequence data represents a promising and effective alternative approach to identify specimens throughout all life stages (Olson et al. 1991, Caterino and Tishechkin 2006, Shank et al. 2006, Bracken-Grissom et al. 2012, Torres et al. 2014 but see Page and Hughes 2011). For animals, mitochondrial DNA (mtDNA) became highly attractive for molecular species identification due to several characteristics: generally high substitution rates, lack of introns, large copy numbers in each cell, and an almost exclusive maternal and haploid inheritance with no recombination (Ballard and Whitlock 2004, Ballard and Rand 2005, Bernt et al. 2013). In this context, a fragment of the mitochondrial cytochrome *c* oxidase subunit I (COI) gene was proposed as so-called ”DNA barcode” for animal species identification more than a decade ago (Hebert et al. 2003a). The efficacy of DNA barcoding is based on a simple assumption: each species will most likely have similar DNA barcodes representing their intraspecific variability whereas the genetic variation between species exceeds the variation within species (Hebert et al. 2003a, 2003b, 2004). In contrast to DNA taxonomy which focuses on the classification of both known and undescribed species based on sequence data only (Tautz et al. 2003, Vogler and Monagham 2007), the central aim of DNA barcoding is two-fold: 1) to assign unknown specimens to already described and classified species, and 2) to enhance the discovery of new species and facilitate identification, particularly in cryptic, microscopic, and other organisms with complex or inaccessible morphology (Hebert et al. 2003a, 2003b). Based on these assumptions, the public Barcode of Life data base (BOLD; www.boldsystems.org) acts as the core data retrieval interface, allowing researchers to collect, manage, and analyze DNA barcode data (Ratnasingham and Hebert 2007). As one of various analytical tools implemented in BOLD, barcodes can be analyzed using the Barcode Index Number (BIN) system (Ratnasingham and Hebert 2013). This approach allows a comparison of specimens identified by morphological and molecular characters.

Not surprisingly, DNA barcoding has been criticized from its beginning. In various cases, DNA barcoding was considered as a useless and expensive identification method (e.g. Will et al. 2005, Cameron et al. 2006, Ebach 2011, Taylor and Harris 2012). Other studies query methodological problems of the analysis of DNA barcodes, for example the inappropriate use of neighbor-joining trees or of fixed distance thresholds (e.g. Will and Rubinoff 2004, Goldstein and DeSalle 2010, Collins and Cruickshank 2013). Finally, another major criticism of this approach was that a single molecular marker such as COI will not necessarily provide sufficient information to deliver the resolution needed to diagnose the large number of species targeted by the initiative (e.g. DeSalle et al. 2005, Prendini 2005, Will et al. 2005). In fact, various aspects can limit the use of COI and mitochondrial DNA in general for successful species delineation. Recent speciation events, heteroplasmy, incomplete lineage sorting as consequence of phylogeographic processes, or the presence of mitochondrial pseudogenes (also known as nuclear mitochondrial DNA or numts) (e.g. Funk and Omland 2003, Bucklin et al. 2011). Furthermore, low evolutionary rates for mitochondrial genes have been demonstrated for various taxa (e.g. anthozoans and some sponges) (e.g. Shearer et al. 2002, Shearer and Coffroth 2008, Sinniger et al. 2008, McFadden et al. 2011).

Nevertheless, DNA barcoding has been successfully applied in a large number of taxonomic groups belonging to both invertebrates (e.g. Carr et al. 2011, Hausmann et al. 2011, Woodcock et al. 2013, Layton et al. 2014, Raupach et al. 2014, Raupach et al. 2015) and vertebrates (e.g. Lijtmaer et al. 2011, Ivanova et al. 2012, Knebelsberger et al. 2014). Furthermore, DNA barcodes have become an integrative part of many recently published species descriptions (e.g. Riedel et al. 2013, Khalaji-Pirbalouty and Raupach 2014, Weis et al. 2014, Hansson et al. 2015).

Within the invertebrates, the Crustacea constitute a challenging taxon for DNA barcoding. With more than 67,000 described species so far (Ahyong et al. 2013), this taxon is species-rich, morphologically diverse and ecologically important. Various crustacean species are of high economic interest (e.g. lobsters, crabs, or shrimps) and represents the basis of extensive crustacean fisheries around the world. Crustaceans can be found in all aquatic environments, and some of them successfully colonized terrestrial habitats in various degrees (e.g. talitrid amphipods, terrestrial crabs, and woodlice). However, a correct identification to the species level is not straightforward for most crustacean taxa, especially for larval and immature stages. Even as adults, numerous species are difficult to identify using morphological characters and usually require the help of taxonomists to differentiate subtle degrees of morphological variability and polymorphism within and between species. This is especially true for small deep-sea crustaceans (e.g. isopods, amphipods and tanaids), and species of the meiofauna (e.g. harpacticoid copepods).

In this review we provide an update regarding the progress of DNA barcoding in crustaceans based on descriptive statistics. Major points of the review are: taxonomic coverage, subject areas, and the use of BOLD as a major platform for the standardization of barcoding studies.

## Methods

This manuscript covers research articles published between 01-01-2003 and 31-12-2014 and available in the “Web of Science” (WoS) database maintained by Thomson Reuters (http://webofknowledge.com). WoS was searched on 15-01-2015 by using “barcod*” and “crusta*” as keywords in the topic of articles hosted by all databases associated with WoS. For comparison purposes, similar searches were conducted for other arthropod taxa on the same day: Insecta (“insect*”), Chelicerata (“chelicer*”) and Myriapoda (“myriapod*”) in combination with “barcod*”. All crustacean references were individually and carefully checked for inconsistencies, in particular false positive results (e.g. articles dealing with other taxa than crustaceans) and duplications. Only publications of the type “article” were kept for further analyses. Language was not selected as filter criterion, and non-English publications with a title and abstract in English were included. Following a strict terminology for DNA barcoding (*sensu* Hebert et al. 2003a), all articles using a different molecular marker than COI-5P’ were excluded. The taxonomic focus was inferred based on the same source (titles, abstracts, keywords) and each article received a label corresponding to one crustacean order with a few exceptions: Calanoida, Harpacticoida, Cyclopoida and Siphonostomatoida were combined into “Copepoda”; Kentrogonida, Scalpelliformes and Sessilia were combined into “Cirripedia”; and the taxon Ostracoda was left at the class level. Articles that covered more than one order and did not fall into the “Copepoda” or “Cirripedia” were classified as “Crustacea”. We used the recent crustacean classification of Ahyong and co-authors (2011) throughout this review as a taxonomic framework. Based on our judgment derived from reading the title, abstract, keywords and, if necessary, portions of articles, we divided all references into six subject areas: 1) identification, library (DNA barcodes used for specimen identification and/or to develop reference barcode libraries), 2) genetic variability (DNA barcodes used for studies on intraspecific genetic variability such as phylogeographic studies), 3) species description (DNA barcodes used together with morphological characters as part of species descriptions), 4) phylogenetics (DNA barcodes used in phylogenetic studies), 5) methods (new lab protocols or new primers developed for barcoding crustaceans), and 6) numts (nuclear mitochondrial DNA sequences and their implications for barcoding crustaceans). In addition and where possible, each article received a label corresponding to the habitat investigated (“marine”: oceans, seas, brackish waters; “freshwater”: rivers, lakes, ponds, groundwater; “mixed”: marine and freshwater). Moreover, geographic labels were assigned to each article based on the main regions covered (continents and oceans). In cases of more than one ocean or continent sampled within the same article, multiple labels were assigned.

In order to verify the popularity and use of the BOLD workbench among crustacean barcoders, each article was searched for referencing BOLD and given a label: ‘YES’ or ‘NO’. If a BOLD project was mentioned by code or title, subsequent steps were followed to find particular records in BOLD and import them into a dataset: 1) search by project code/title in BOLD Workbench, 2) copy all records from that project, and 3) add records to dataset. All public records stored in BOLD and generated by crustacean barcoding studies can be retrieved by searching DS-CRST (Title: Crustacean Barcoding Studies) in BOLD or by going directly to the corresponding DOI associated with this dataset (http://dx.doi.org/10.5883/DS-CRST). By using a project code as search term, all records of that project were imported, regardless of its history (i.e., records added or removed from a project) between the publication date and January 2015). Some articles mentioned the use of BOLD without providing a project code. In such cases, we were able to find records by the process IDs mentioned in the publication or by searching BOLD based on taxa names. However, when tracking records was not a straightforward process, we excluded those studies from our BOLD-related analyses. DS-CRST in BOLD was used for standard barcoding analyses: number of species versus number of BINs, taxon ID tree and distance summary. Geographic coordinates, where available, were exported and used to create a map in QGIS (QGIS Development Team 2015).

Additional bibliographic data were compiled for all references: publication title, first authors’ names, journal name, publication year, open-access feature, and the number of citations (as provided by WoS). The major results of our literature review are summarized graphically; a table containing all raw data is available as Suppl. material 1.

## Results

**Figure 1.**
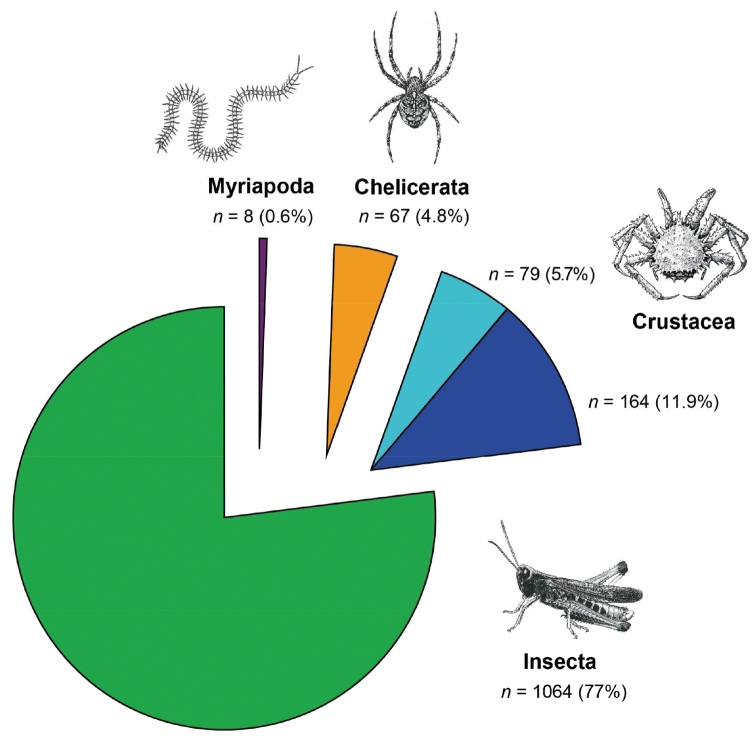
DNA barcoding studies of the Arthropoda. Total number and percentage values of articles published with “barcod*” and insect*” (green), “crusta*” (blue), “chelicer*” (orange), or “myriapod*” (violet) as keywords in their topic and listed in the Web of Science (period covered: 2003-2014; *n* = 1,382). For crustaceans, the total number of articles is split into: 1) the number of articles removed from our analysis (duplications and false positives) (pie sector in light blue) and 2) the core number of articles used in this review (pie sector in dark blue). Arthropod illustrations were modified from Gruner (1993) and Dathe (2003).

**Figure 2.**
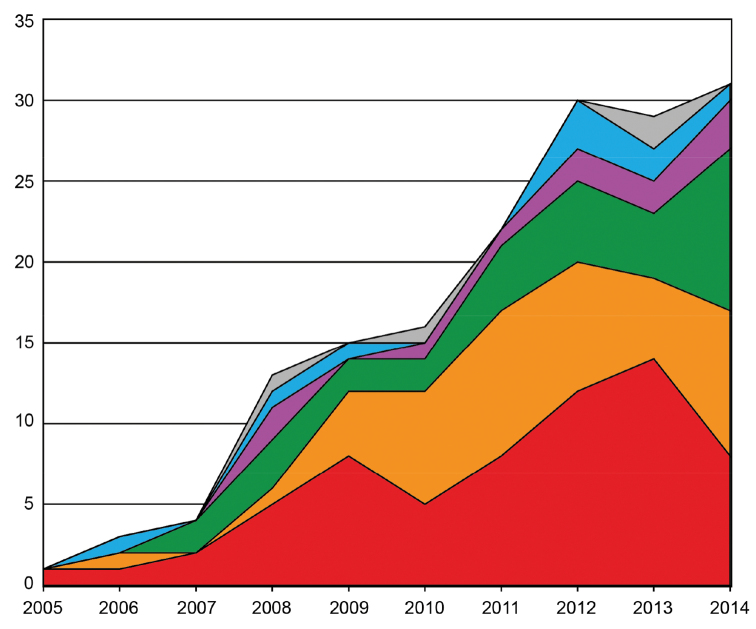
Subject areas of DNA barcoding studies of the Crustacea. Number of articles with “barcod*” and “crusta*” as keywords in their topic as retrieved from the Web of Science (period covered: 2003–2014; *n* = 164) and divided into six subject areas (from bottom to top): identification and barcode library (red), genetic variability (orange), species description (green), phylogenetics (violet), methods (blue), and numts (grey).

Our search in WoS produced 243 hits associated with the terms “barcod*” and “crusta*”, 1,064 references for “barcod*” and “insect*”, 67 for “barcod*” and “chelicer*” and eight for “barcod*” and “myriapod*” (Fig. 1). In total, 1,382 publications were found for all Arthropoda. Our initial list of 243 crustacean references was revised and reduced to 164 publications after removing duplicates and mislabeled references. All other arthropod references were not revised in detail. The number of barcoding publications showed a fast increase from the first and singular crustacean article published in 2005 (Page et al. 2005) up to 30 publications in 2012 (Fig. 2). In 2013, a slight decrease to 29 publications was observed, followed by an increase to 31 publications in 2014. However, the frequencies of the different categories fluctuated each year (Fig. 2).

The taxonomic coverage of the 164 barcoding publications showed a strong preference for the Decapoda (*n* = 60, 36.7%), followed by the mixed taxon of “Crustacea” (*n* = 28, 17%), the Amphipoda (*n* = 21, 12.8%), Copepoda (*n* = 18, 11%), and Diplostraca (*n* = 13, 8%) (Table 1). All other crustacean taxa have been investigated by less than ten publications: Isopoda (*n* = 6, 3.7%), Anostraca and Cirripedia (*n* = 5, 3%), Stomatopoda (*n* = 3, 1.8%), and Bathynellacea (*n* = 2, 1.2%). The Euphausiacea, Ostracoda, and Tanaidacea have been analyzed only once (each with *n* = 1, 0.6%).

**Table 1.** Number of publications of the Crustacea using DNA barcodes. “Barcod*” and “crusta*” were used as keywords in the Web of Science (2003–2014). For comparison, the most recent species count per taxon is given in a separate column (based on Ahyong et al. 2011).

Taxon		Publications	(%)	Number of described species
**Malacostraca**	Decapoda	60	36.7	14,895
	Amphipoda	21	12.8	9,896
	Isopoda	6	3.7	10,661
	Stomatopoda	3	1.8	460
	Bathynellacea	2	1.2	241
	Euphausiacea	1	0.6	87
	Tanaidacea	1	0.6	1,069
**Maxillopoda**	Copepoda	18	11	15,976
	Cirripedia	5	3	1,306
**Branchiopoda**	Diplostraca	13	8	821
	Anostraca	5	3	313
**Ostracoda**		1	0.6	7,577
“**Crustacea**“		28	17	n. a.
**Total**		**164**	**100**	

Our investigation also revealed that most crustacean barcoding studies focus on the identification of specimens and the expansion of reference libraries for various taxa (*n* = 64, 39.1%) (Table 2). Beside identification, DNA barcodes were frequently used in publications analyzing the genetic variability of species (*n* = 44, 26.8%) and as additional characters in species descriptions (*n* = 32, 19.5%). Relatively small numbers of publications covered the use of DNA barcodes as part of phylogenetic reconstructions (*n* = 11, 6.7%), the publication of new protocols and methods to obtain barcode sequences (*n* = 9, 5.5%), and the study of numts (*n* = 4, 2.4%).

**Table 2.** Subject area and taxonomic rank of DNA barcoding studies of the Crustacea. Number of articles were retrieved by using “barcod*” and “crusta*” as keywords in the topic of articles hosted by the Web of Science (period covered: 2003–2014).

	Identification, library	Genetic variability	Species description	Phylogenetics	Methods	numts
**Decapoda**	26	11	15	5	1	2
**Amphipoda**	4	15	1	1		
**Isopoda**		2	3		1	
**Stomatopoda**	3					
**Bathynellacea**		1	1			
**Euphausiacea**	1					
**Tanaidacea**	1					
**Copepoda**	4	5	6	3		
**Cirripedia**	2		2	1		
**Diplostraca**	2	8	3			
**Anostraca**	1	1	1	1	1	
**Ostracoda**	1					
“**Crustacea**”	19	1			6	2
**Total**	**64**	**44**	**32**	**11**	**9**	**4**

**Figure 3.**
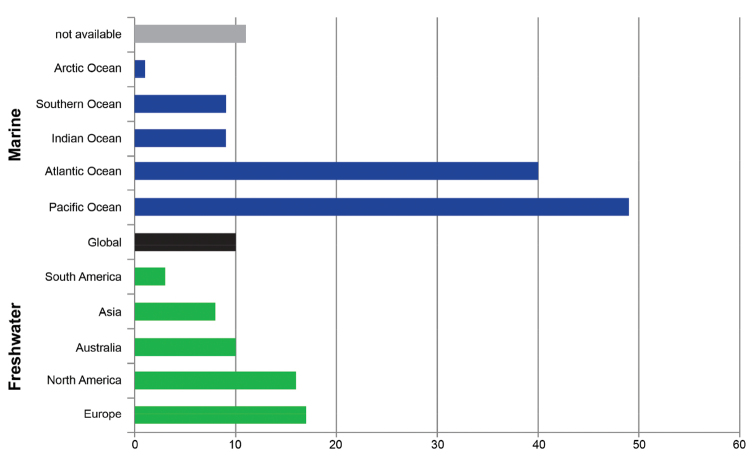
Geographic and habitat focus of the analyzed DNA barcoding studies of the Crustacea. Studies were listed in the Web of Science (period covered: 2003–2014, *n* = 164), with the number of publications shown on the X axis. Green bars indicate freshwater studies, dark blue bars marine studies. A black bar represents studies that were performed on a global scale. For 11 studies, no classification was possible (grey bar). Note that publications can include taxa from more than one habitat or region.

Approximately two thirds of the barcoding studies focused on the marine environment (*n* = 99, 60.4%) and only one third dealt with freshwater systems (*n* = 49, 29.8%) (Fig. 3). Six studies covered taxa from both marine and freshwater habitats (*n* = 6, 3.7%), and for ten studies no classification was possible (6.1%). Interestingly, no study was found analyzing terrestrial crustaceans exclusively (e.g. woodlice) (Suppl. material 1). Our geographic investigation covered only the major divisions of land and water, namely continents and oceans. It should be noted that publications can include taxa from more than one environment or geographic region. The analyzed DNA barcoding publications covered all oceans (the Arctic, Atlantic, Indian, Pacific, and Southern Ocean), with a focus on the Pacific Ocean (*n* = 49, 25.5%), followed by the Atlantic Ocean (*n* = 28, 14.5%) (Fig. 3). In the case of continents, five were sampled: Asia (*n* = 8, 4.2%), Australia (*n* = 10, 5.2%), Europe (*n* = 17, 8.9%) as well as North and South America (*n* = 17, 8.9%; *n* = 3, 1.6%) (Fig. 3). Ten studies (5.2%) had a global geographic coverage, whereas it was impossible to place the origin of the specimens analyzed for 11 studies (5.7%), e.g. studies which used data mined from GenBank (Suppl. material 1).

**Figure 4.**
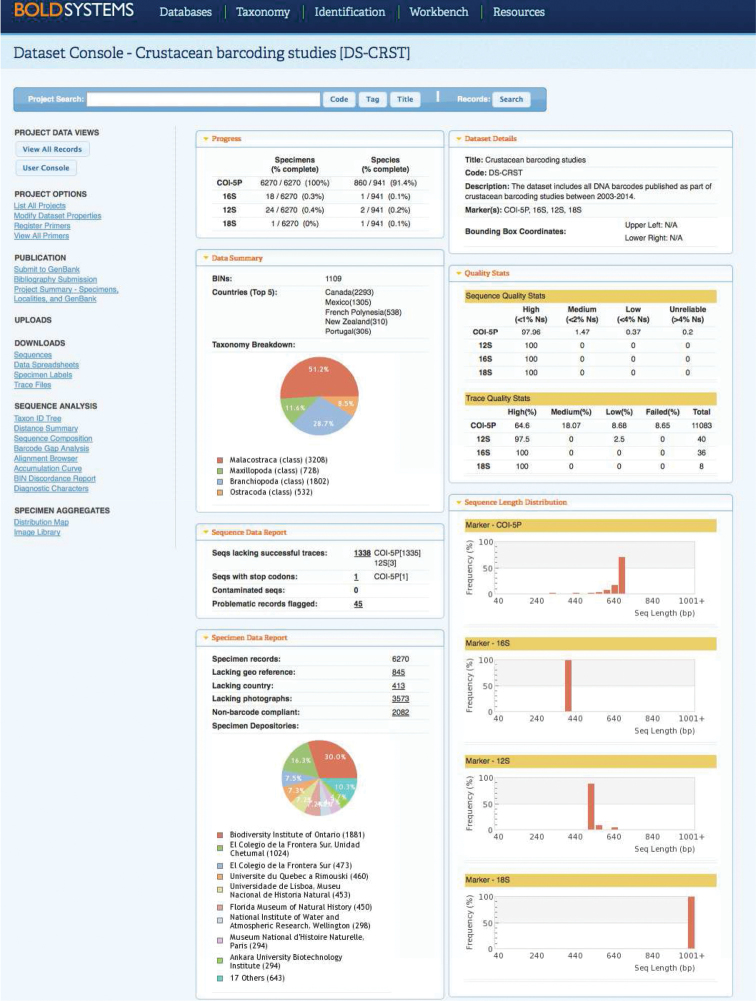
Project console for DS-CRST in BOLD. Various statistics for the current status of specimens are displayed: record count, species count, taxonomy breakdown, specimen depositories, country of collection, sequence count, flagged records count, trace count, image count. Note that BOLD is a dynamic environment and updates will be reflected on the project console.

**Figure 5.**
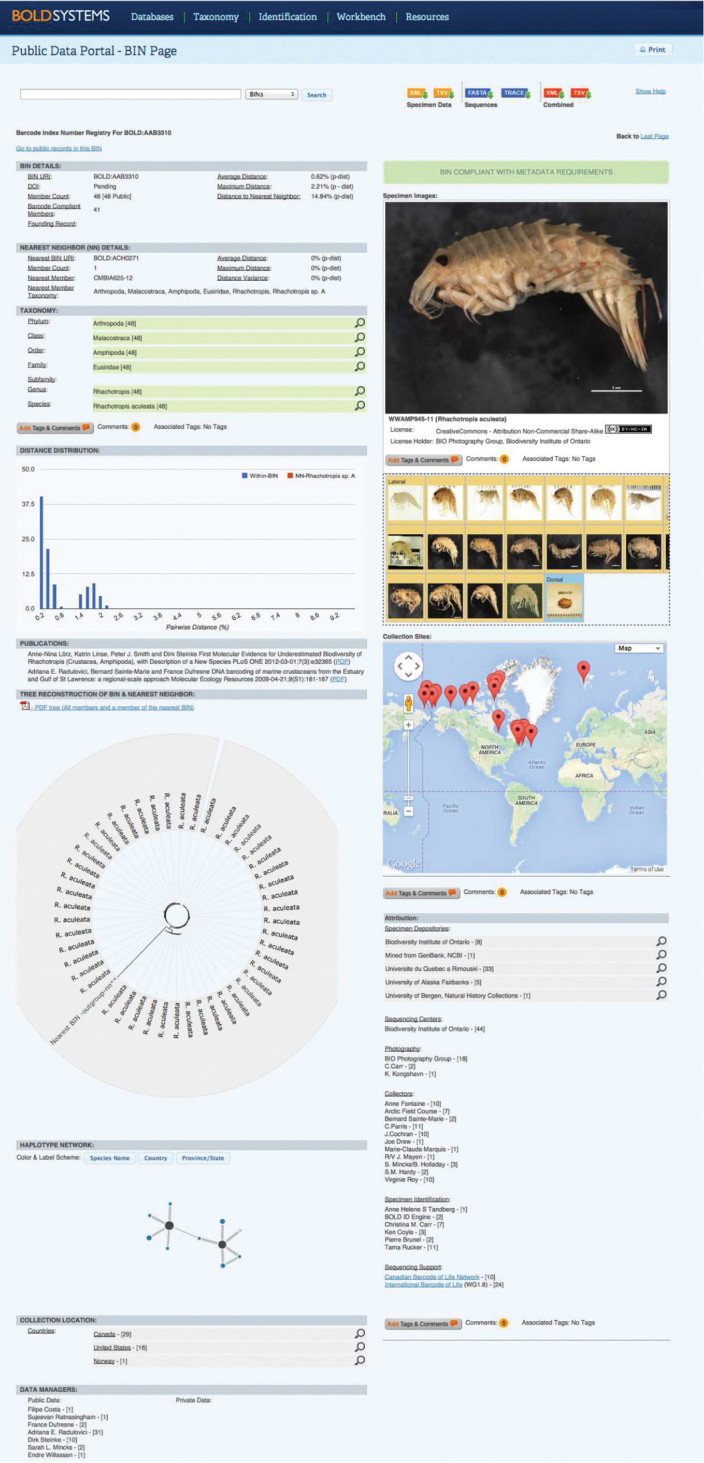
Example for a BIN page in BOLD. The amphipod *Rhachotropis aculeata* (Lepechin, 1780) is registered in the BIN registry as BOLD:AAB3310. Note that BOLD is a dynamic environment and updates will be reflected on the BIN page, including BIN changes.

**Figure 6.**
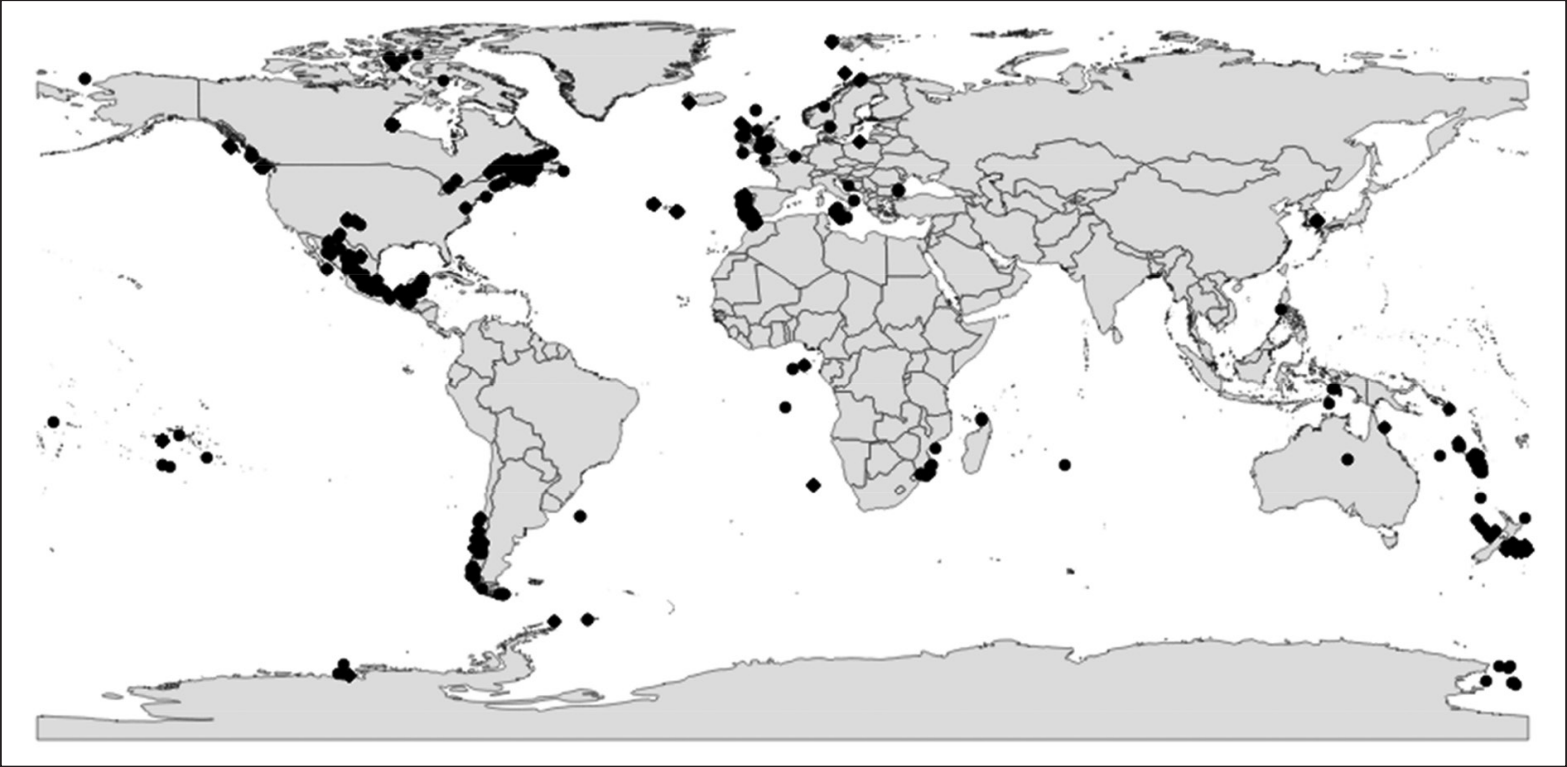
Sampling locations for crustaceans used in this review. GPS data was taken from the dataset DS-CRST in BOLD. Note that only 5,425 out of 6,270 records had GPS coordinates and are reflected here.

The vast majority of publications (*n* = 129, 78.7%) did not mention BOLD in their text (label ‘NO’ in Suppl. material 1). The remaining 35 publications (21.3%) used BOLD as part of their study with project titles/codes (*n* = 23, 14%), or with projects created *a posteriori*, similar to the workflow of sequence publication in GenBank (*n* = 3, 1.8%). A handful of articles used BOLD exclusively for data mining or as an identification engine for DNA sequences or mentioned BOLD as part of current or proposed DNA barcoding workflows. A total of 6,270 records were successfully tracked and imported into DS-CRST (Fig. 4). Approximately half of the records belonged to Malacostraca (*n* = 3,208, 51.2%), followed by Branchiopoda (*n* = 1,802, 28.8%), Maxillopoda (*n* = 728, 11.6%), and Ostracoda (*n* = 532, 8.5%). In total, 5,740 records (91.5%) had species names (Linnaean names or interim names) while 530 crustaceans (8.5%) remained unidentified (March 2015). Data owners inserted 860 species names whereas BOLD assigned 1,109 BINs to the entire dataset (Fig. 5). Furthermore, 413 records (6.6%) lacked details about the country of sample collection, 845 records (13.5%) lacked GPS coordinates whereas 3,573 specimens (57%) provided no image for the voucher. Records with collection details were divided between Canada (*n* = 2,293, 36.6%) and Mexico (*n* = 1,305, 20.8%) plus another 38 countries with much fewer records (Fig. 6). In addition to 6,270 DNA barcodes, some records used supplementary genetic markers (12S, 16S, and/or 18S rDNA). A number of 1,338 records (21.3%) had no successful chromatogram (“trace”) associated, one COI sequence (0.02%) had stop codons and 45 records (0.7%) had been flagged as misidentification or contamination between the publication date and March 2015. A total of 2,082 records (33.2%) were non-barcode compliant (i.e., one of the following criteria was not fulfilled: country, two trace files, a fragment length of at least 500 base pairs, and less than 1% ambiguities).

We found 76 different journals publishing articles dealing with DNA barcoding and crustaceans. Most studies were published in Zootaxa (*n* = 23, 14%), followed by the Journal of Crustacean Biology and PLOS ONE (each with *n* = 9, 5.5%), Molecular Ecology Resources (*n* = 7, 4.3%), Crustaceana and Invertebrate Systematics (each with *n* = 6, 3.7%). A number of 50 journals (65.8%) had only one article dealing with crustacean barcoding. Only 33 articles (20.1%) were open access as they were published in open access journals (e.g. PLOS ONE, ZooKeys) or in subscription journals where authors chose to publish their work as open-access (Suppl. material 1). The author list revealed a total number of 700 authors with 125 being first authors. The most prolific first author of crustaceans and DNA barcodes was Arthur Anker (7 articles in total, 4.3%), followed by Tomislav Karanovic (4 articles, 2.4%) and Ann Bucklin, Manuel Elías-Gutiérrez, Laetitia Plaisance, and Chien-Hui Yang, each with three first-authored papers involving DNA barcoding of crustaceans. The most cited article by far was written by Song and co-authors (2008) discussing the effects of numts for DNA barcoding (292 citations), followed by a publication of Lefébure and co-authors (2006) discussing threshold calculations for a successful species identification (185 citations), Witt and co-authors (2006) with one of the first articles on the role of DNA barcoding in highlighting the existence of cryptic species (172 citations), and Costa and co-authors in 2007 with the first comprehensive study testing the efficacy of DNA barcoding for crustacean species identification (165 citations) (Table 3). In the case of phylogenetic analyses using DNA barcode data the most cited article was published by Matzen da Silva and co-authors (2011a), focusing on the Malacostraca (21 citations). Finally, Lai and co-authors (2010) included DNA barcodes in their revision of the *Portunus pelagicus* (Linnaeus, 1758) species complex. This article was cited 23 times.

**Table 3.** Most cited crustacean barcoding articles per subject area. Data obtained from Web of Science based on a query with ‘barcod*’ and ‘crusta*’ as keywords in the topic of articles published between 2003 and 2014. Citations are given as the total number of citations since publication and the average number of citations per year (in brackets).

Subject area	Title	Authors	Journal	Year	Citations
Identification, library	Biological identifications through DNA barcodes: the case of the Crustacea	Costa FO, deWaard JR, Boutillier J, Ratnasingham S, Dooh RT, Hajibabaei M, Hebert PDN	Canadian Journal of Fisheries and Aquatic Sciences	2007	165 (18.3)
Genetic variability	DNA barcoding reveals extraordinary cryptic diversity in an amphipod genus: implications for desert spring conservation	Witt JDS, Threloff DL, Hebert PDN	Molecular Ecology	2006	172 (17.2)
Species description	A revision of the *Portunus pelagicus* (Linnaeus, 1758) species complex (Crustacea: Brachyura: Portunidae), with the recognition of four species	Lai JC, Ng PKL, Davie PJF	Raffles Bulletin of Zoology	2010	23 (3.8)
Phylogenetics	Systematic and evolutionary insights derived from mtDNA COI barcode diversity in the Decapoda (Crustacea: Malacostraca)	Matzen da Silva J, Creer S, dos Santos A, Costa AC, Cunha MR, Costa FO, Carvalho GR	Public Library of Science ONE	2011	21 (4.2)
Methods	Relationship between morphological taxonomy and molecular divergence within Crustacea: proposal of a molecular threshold to help species delimitation	Lefébure T, Douady CJ, Gouy M, Gibert J	Molecular Phylogenetics and Evolution	2006	185 (18.5)
numts	Many species in one: DNA barcoding overestimates the number of species when nuclear mitochondrial pseudogenes are coamplified	Song H, Buhay JE, Whiting MF, Crandall KA	Proceedings of the National Academy of Sciences of the USA	2008	292 (36.5)

## Discussion

During the past few years, crustaceans have become a popular target for DNA barcoding among the Arthropoda, being outnumbered only by barcoding studies of the Insecta (Fig. 1). Although the observed ratio of barcoding articles of insects compared to barcoding publications for crustaceans is high (6:1), this is not surprising since insects represent the most species-rich taxon on earth (app. 1 million species described and app. 5 million species estimated) (Chapman 2009). Crustacean publications showed a continuous increase starting with the first publication in 2005. In recent years, the numbers of crustacean publications seem to have reached a plateau with approximately 30 publications per year.

Although we used a highly popular database which indexes scientific literature, we are aware that an unknown number of references are missing from our study. This is mainly caused by two reasons: 1) the term “DNA barcoding” was not used in the publication although COI sequences were applied for species identification (e.g. Shih and Cai 2007), and 2) some journals might not be indexed in WoS yet. Despite this somewhat incomplete sampling of literature, we think that our review reflects the application of DNA barcodes in carcinology in a representative way.

### Taxonomic overview

A rapid investigation of the taxonomic diversity covered in the 164 barcoding publications showed the highest frequency for Malacostraca (*n* = 94, 57.4%), the class with the largest number of crustacean species (Ahyong et al. 2011, Appeltans et al. 2012) and the most familiar ones (e.g. lobsters, crabs, shrimps, krill, beach hoppers, woodlice). Within the Malacostraca, the ecologically and economically important Decapoda were most popular for barcoding studies (*n* = 60 articles, 36.7%), followed by the Amphipoda (*n* = 21, 12.8%), a species-rich group inhabiting most aquatic habitats and even some terrestrial habitats with high humidity (e.g. supralittoral, rainforests) (Table 1). Other malacostracan orders seem to be less popular for specific DNA barcoding despite high or moderate numbers of known species, e.g. the Isopoda (10,000 species, 6 publications, 3.7%) or Tanaidacea (1,000 species, 1 publication, 0.6%). So far, no study focused specifically on the Cumacea (1,500 species) or Mysida (app. 1,200 species). We hope that scientists working on these taxa become more aware of the benefits of DNA barcoding as part of their studies, inducing an increase in the number of publications in the near future. The Maxillopoda, the second most species-rich crustacean class representing much of the marine and freshwater zooplankton, was covered in 23 studies (14%). Copepods were most popular among the maxillopods (*n* = 18, 11%), as it can be expected for a species-rich group (app. 16,000 species) with ecological importance in planktonic food-webs, as opposed to Cirripedia covered by only five publications (3%). The third most popular crustacean class was the Branchiopoda (*n* = 18, 11%), a group of crustaceans frequently encountered in freshwater habitats. Surprisingly, the species-rich class of Ostracoda (app. 7,500 species) has been covered as an exclusive taxon in only one publication (0.6%) until now. Furthermore, 28 publications (17%) had a mixture of different taxonomic groups (i.e. multiple orders were sampled) and were labeled as “Crustacea”. These were usually subject-oriented (e.g. reviews on various topics) rather than taxon-oriented publications. The remaining two classes of crustaceans, Remipedia and Cephalocarida, have not been targeted by DNA barcoding studies yet (January 2015). A search using the taxon names and “COI” in GenBank returned 24 hits for the Remipedia and 20 for the Cephalocarida. Not surprisingly, these species-poor taxa (Remipedia: 18 species, Cephalocarida: 13 species; Ahyong et al. 2011) are also less important from an economic or ecological perspective. Although we do not expect comprehensive barcoding studies for species-poor taxa in the near future, we believe they might be targeted as part of comprehensive regional studies.

### Subject areas of DNA barcoding publications

In contrast to the total number of publications, which revealed a steady increase followed by a relative plateau, the trend for the six subject areas (see methods) showed large fluctuations from year to year (Fig. 2). Overall, our analyses revealed that most barcoding studies focused on species identification linked to building or expanding existing reference libraries of COI sequences (*n* = 64, 39.1%), followed by analyses of the intraspecific genetic variability (*n* = 44, 26.8%) and by species descriptions that use DNA barcodes as additional characters (*n* = 32, 19.5%) (see Table 2). Less common were studies using DNA barcodes in molecular phylogenetics (*n* = 11, 6.7%), new methods and protocols (*n* = 9, 5.5%) or the possible effects of numts for barcoding studies of crustaceans (*n* = 4, 2.4%). We provide more details for each subject area in the following paragraphs.

### Species identification and DNA barcode libraries

Species identification based on DNA barcodes relies on the existence of reference libraries which consist of COI sequences from specimens previously identified by experts based on traditional methods (i.e., morphological characters). Consequently, many barcoding studies published so far deal with the development of comprehensive barcode libraries (e.g. Dincǎ et al. 2010, Baird et al. 2011, Zhou et al. 2011, Raupach et al. 2014, Rougerie et al. 2014) and their use to identify unknown specimens (e.g. Holmes et al. 2009, Strutzenberger et al. 2011, Shen et al. 2013, Knebelsberger et al. 2014). Similar to this general trend, most crustacean publications reviewed here were found to fit in this category (*n* = 64, 39.1%), with a constant increase over the years (Fig. 2). In terms of crustacean diversity, most studies were performed on the Decapoda (*n* = 26, 40.6%) and the mixed group of “Crustacea” (*n* = 19, 29.9%). All the other crustacean taxa were investigated by less than five publications each (Table 2). A constantly growing library of DNA barcodes will offer numerous applications, such as seafood traceability (e.g. Haye et al. 2012, Nicolè et al. 2012, Di Pinto et al. 2013), the identification of larvae (e.g. Barber and Boyce 2006, Webb et al. 2006, Weigt et al. 2012), and tools for ecological studies in general (e.g. Valentini et al. 2009, Bowser et al. 2013, Burghart et al. 2014). Moreover, comprehensive barcode libraries will become essential for biomonitoring applications based on modern high-throughput sequencing technologies (e.g. Fonseca et al. 2010, Hajibabaei et al. 2011, Shokralla et al. 2012, Thomsen et al. 2012, Zhou et al. 2013, Leray and Knowlton 2015).

### DNA barcodes and intraspecific genetic variation

The study of intraspecific genetic variation in relation to geography has become very popular in recent decades and resulted in the formation and expansion of a new research field, namely phylogeography (Avise 2000, Hickerson et al. 2010). In the past, numerous phylogeographic studies have been published on various taxa, including crustaceans (e.g. Audzijonyte et al. 2006, Krebes et al. 2010, Campo et al. 2010, Garcia-Merchan et al. 2012, Santamaria et al. 2013). The body of sequence data generated through such phylogeographic studies was actually the background on which DNA barcoding was proposed as a method for species identification across the entire animal kingdom (Hebert et al. 2003a, 2003b). As COI sequences are used in DNA barcoding as well as in phylogeography, it is no surprise that publications with “barcod*” and “crusta*” as keywords investigate the level of genetic diversity within species as well (Fig. 2). Our review identified 44 studies for this category. Interestingly, the amphipods (*n* = 15, 34.1%) were more popular than decapods (*n* = 11, 25%) for this subject area. All other crustacean groups were present in less than ten publications per taxon (Table 2). To verify the progress in crustacean phylogeographic studies, we used phylogeograph*”, “crusta*” and “cytochrome oxidase I” as keywords in WoS and retrieved 152 articles. The large discrepancy between our review and WoS is caused by the fact that the term “DNA barcode” is normally not used in phylogeographic studies as keyword. However, the variation of intraspecific genetic diversity in relation with spatial scales may have an important impact on the efficacy of DNA barcoding (Bergsten et al. 2012). Therefore we encourage researchers interested in phylogeography to address problems related to DNA barcoding as well.

### New species description including DNA barcodes

Ideally, DNA barcoding and species discovery would be seen as intertwined. Whereas the main objective of DNA barcoding is to identify unknown specimens based on reference libraries, an additional outcome is reflected in the identification of unknown genetic clusters that might represent new species. As such, DNA barcodes represent powerful diagnostic supplementary characters that accelerate and revive traditional morphological taxonomy but do not replace it (DeSalle et al. 2005). It is not surprising that more and more species descriptions include barcode sequences or that entire monographs are triggered by the results of DNA barcoding (Butcher et al. 2012, Landry et al. 2013). In total, we found 32 publications incorporating DNA barcodes as part of new species descriptions of crustaceans (Table 2, Fig. 2). Again, the Decapoda were the dominant taxon (*n* = 15, 46.9%). Other studies focused on Copepoda (*n* = 6, 18.8%), Diplostraca and Isopoda (each with *n* = 3, 9.4%), Cirripedia (2, 6.2%), and the Amphipoda, Anostraca, and Bathynellacea (each with *n* = 1, 3.1%). In this context we used Thomson Reuter’s Zoological Record through the Index of Organism Names (www.organismnames.com) to calculate the rate of crustacean species descriptions during the last decade. The Metrics function and the “Graphs of new taxa over time” option showed a fluctuating rate between 681 (minimum in 2014) and 1,263 (maximum in 2008) with a mean of 891 new crustacean species being described each year, with one third representing decapods. This large discrepancy between the numbers of new species being described per year and the numbers of studies implementing DNA barcoding for species description (278:1) reflects the hesitation of taxonomists to adopt new approaches on large scale or their limited access to sequencing technologies. We hope for a change of mentality in the near future and an increased access to molecular labs as a combination of morphological and molecular data allows more detailed species descriptions as part of an aspired integrative taxonomy (e.g. Dayrat 2005, Padial et al. 2010, Schlick-Steiner et al. 2010). In addition, the new approach would also include a standardized analytical package: raw distance data (percent divergence), diagnostic characters and phylogenetic trees (Goldstein and DeSalle 2010).

### DNA barcodes and phylogenetic analyses

During the last years, COI sequences combined with other mitochondrial and nuclear markers have been frequently used to reconstruct the phylogeny of various taxa of the Crustacea (e.g. Blanco-Bercial et al. 2011, Matzen da Silva et al. 2011b, Klaus et al. 2013). Similar to phylogeographic studies, the term DNA barcode is typically not used in this context. Nevertheless, we found 11 publications using the term DNA barcodes as part of molecular phylogenetic studies, with five studies analyzing relationships of the Decapoda (45.4%), three references for the Copepoda (27.3%), and one reference for the Amphipoda, Anostraca and Cirripedia (each 9.1%), respectively (Table 2). Whereas DNA barcodes may be useful to reconstruct recent radiations and/or speciation events in some cases (e.g. Schubart et al. 1998, Cristescu and Hebert 2002), the combination of mitochondrial DNA with more conserved nuclear markers (e.g. 18S or 28S rRNA genes) is essential when reconstructing higher taxa phylogenies (Schubart 2009).

### Laboratory protocols and methods

Although DNA barcoding as a molecular method for species identification has been in use for more than a decade, techniques for generating, applying, and analyzing barcode data are still being improved to guarantee an efficient workflow (e.g. Lopez and Erickson 2012). We found nine studies presenting new protocols for DNA extraction or newly designed primer pairs for crustaceans. Six publications focused on various taxa of the “Crustacea” (66.7%), and one publication for each of the remaining taxa: Anostraca, Decapoda and Isopoda (each 11.1%). As DNA barcoding becomes more and more accepted in carcinology, we are convinced that the development of more specialized protocols as well as the optimization of taxa-specific primer pairs will increase in the near future (e.g. Schubart 2009), making DNA barcoding easier and more popular for carcinologists.

### Nuclear copies of mitochondrial DNA: numts

The unwanted amplification of nuclear copies of mitochondrial DNA (numts) represents a problem not only for the analyses of DNA barcodes (COI sequences) but mitochondrial genes in general (Bensasson et al. 2010, Hazakani-Covo et al. 2010). Whereas numts can be useful for phylogenetic or population structure analyses in some special cases (Pons and Vogler 2005, Hazakani-Covo 2009, Soto-Calderón et al. 2014), their presence may represent a serious problem for barcode studies. Numts are known for various taxa, including mammals (e.g. Thalmann et al. 2005, Kim et al. 2006, Soto-Calderón et al. 2014), insects (e.g. Pons and Vogler 2005, Pamilo et al. 2007, Ruiz et al. 2013, Song et al. 2014), as well as crustaceans (e.g. Schneider-Broussard and Neigel 1997, Williams and Knowlton 2001, Buhay 2009, Baeza and Fuentes 2013). Until January 2015, only four studies highlighted the potential issues of numts for DNA barcoding studies of the Crustacea, with a focus on decapods (*n* = 2, 50%) and the mixed “Crustacea” (*n* = 2, 50%). Whereas most numts were found within decapods, it is actually unclear if such pseudogenes may become problematic for other crustacean taxa too. In order to minimize the risks caused by numts for DNA barcoding studies we recommend rigorous quality control of all barcode sequences. This includes a strict use of high-quality chromatograms, a translation of the barcode sequences to amino acids to detect insertions, deletions and/or in-frame stop codons, and the use of taxa-specific primers for some groups (see Song et al. 2008, Schubart 2009).

### Crustacean DNA barcoding and BOLD

In March 2015, the Public Data Portal of BOLD was hosting more than 80,000 DNA barcodes representing about 5,700 crustacean species (plus a large amount of unidentified specimens) and 10,000 BINs. Only 8% (6,270 records; 860 species names) were directly associated with crustacean barcoding studies (35 publications, Suppl. material 1) as the respective authors used BOLD for their research. The remaining crustacean barcodes were associated with private projects and with published sequences mined from GenBank. By retrieving COI data from GenBank that were generated as part of non-barcoding studies but fulfill the ‘barcode’ requirements, BOLD is assembling all information pertaining to reference libraries in a single database, thus reducing the risk of duplication in barcoding the same taxa multiple times. Despite a decade of work in the field of DNA barcoding, only app. 7,000 crustacean species have been barcoded to date (public and private data, available from the Taxonomy Browser in BOLD). However, existing biodiversity catalogues specify a number of more than 67,000 crustacean species described worldwide (Ahyong et al. 2011) and app. 150,000 undescribed species (Chapman 2009), although recent inventories give estimate numbers as high as 200,000–360,000 species in the marine environment alone (Appeltans et al. 2013). In times of limited taxonomic expertise as well as resources and rampant accumulation of barcode data, the option of using a DNA-based registry (such as the BIN system) for crustacean diversity has clear advantages. A fast and accurate clustering of COI sequences into groups corresponding to presumptive species (BINs) would assist in screening large amounts of data and highlighting those cases that need detailed investigation (e.g. taxonomic synonymy, cryptic diversity, specimen misidentification). For instance, 10,000 BINs are available for crustaceans in BOLD, and a rapid initial investigation would require morphological identification of roughly 10,000 specimens as opposed to 80,000 screened through DNA sequencing. Besides identifying cohesive genetic clusters, the BIN system provides a persistent catalogue of biodiversity as each BIN has a unique alphanumeric identifier. In addition, each BIN has an individual webpage in BOLD which displays all the available information: BIN member count, nearest neighbour, genetic distance summary, haplotype network, images, sampling map, specimen depositories, collectors, identifiers, data status (public or private), data owners, annotations inserted by the barcoding community and publications using a specific BIN (Fig. 5). Multiple options to download specimen and/or sequence data are also given.

A growing database such as BOLD, which follows specific high standards for data quality, will certainly be useful for large-scale analyses in crustacean phylogeography, biogeography and biodiversity assessment and will offer support for technological advances such as high-throughput sequencing.

## Conclusions

Our review shows that DNA barcoding has gained popularity in carcinology and that the most popular group targeted for various related topics are the malacostracan crustaceans, in particular decapods. As the main goal of DNA barcoding is to assign unknown specimens to known species, most crustacean barcoding studies were found to build or use existing reference libraries for identification purposes and this trend will surely continue and probably increase in the future. The generation of comprehensive barcode libraries will represent a challenging but also an important task, especially for some species-rich habitats (e.g. the deep sea or coral reefs), where our general knowledge about crustacean diversity, in particular species numbers, is still poor. A second objective of DNA barcoding is to accelerate species discovery, particularly in cryptic, microscopic and other organisms with complex or inaccessible morphology. We believe that more progress will be made in this direction as well.

Crustacean taxonomy seems to be slowly incorporating DNA barcoding in the field as the top journal in this field is a taxonomic journal and the most prolific first authors have a taxonomic background. However, a larger acceptance and application is highly desirable, and therefore we encourage a stronger cooperation between “classical” taxonomists and the DNA barcoding community. Moreover, the term “DNA barcode” should only be used for COI-5P` sequences (Hebert et al. 2003a). In this context we also recommend the use of BOLD for data storage, analysis and publication. By following such standards in data generation and analysis, large comparisons across taxonomic groups would be easily drawn for better predictions of biodiversity, in particular molecular, patterns and species diversity in general.

## Supplementary materials

Supplementary material 1 A decade of DNA barcoding of crustaceans: input file Data type: data table Explanation note: Raw data related to 164 publications on crustacean barcoding as retrieved from Web of Science: bibliography, citations, habitat type, geographical area, BOLD use. This dataset is made available under the Open Database License (http://opendatacommons.org/licenses/odbl/1.0/). The Open Database License (ODbL) is a license agreement intended to allow users to freely share, modify, and use this Dataset while maintaining this same freedom for others, provided that the original source and author(s) are credited.Michael J. Raupach, Adriana Radulovici
